# Inhibitory Effect of Human Anti-CA I Autoantibodies and Development of Monoclonal Antibody mAb 2B8 Targeting Carbonic Anhydrase I

**DOI:** 10.1155/mi/9981131

**Published:** 2024-12-30

**Authors:** Petra Chalova, Barbora Jankovicova, Veronika Dvorakova, Eliska Zelinkova, Zuzana Bilkova, Marcela Slovakova, Lucie Korecka, Petr Muller, Maksym Danchenko, Lenka Minichova, Jan Lakota, Ludovit Skultety

**Affiliations:** ^1^Biomedical Research Center, Slovak Academy of Sciences, Bratislava, Slovakia; ^2^Department of Pharmaceutical Analysis and Nuclear Pharmacy, Faculty of Pharmacy, Comenius University, Bratislava, Slovakia; ^3^Department of Biological and Biochemical Sciences, Faculty of Chemical Technology, University of Pardubice, Pardubice, Czech Republic; ^4^Regional Centre for Applied Molecular Oncology, Masaryk Memorial Cancer Institute, Brno, Czech Republic; ^5^Institute of Chemistry, Slovak Academy of Sciences, Bratislava, Slovakia; ^6^Institute of Normal and Pathological Physiology, Centre of Experimental Medicine, Slovak Academy of Sciences, Bratislava, Slovakia; ^7^Institute of Microbiology, Czech Academy of Sciences, Prague, Czech Republic

**Keywords:** antitumor immune response, carbonic anhydrase I, epitope mapping, esterase activity, inhibitory effect

## Abstract

Spontaneous tumor regression is a recognized phenomenon across various cancer types. Recent research emphasizes the alterations in autoantibodies against carbonic anhydrase I (CA I) (anti-CA I) levels as potential prognostic markers for various malignancies. Particularly, autoantibodies targeting CA I and II appear to induce cellular damage by inhibiting their respective protein's catalytic functions. Our study illuminates the profound impact of anti-CA I autoantibodies from patient serum on the esterase activity of human CA I, exhibiting inhibitory effects akin to the acetazolamide inhibitor. Concurrently, our newly synthesized mouse monoclonal IgG antibody, mAb 2B8, against human CA I showcased a potent inhibitory action. An in-depth exploration into mAb 2B8′s binding dynamics with its target enzyme was undertaken. Leveraging epitope extraction and phage display library techniques, we identified the amino acid sequence DFWTYP (positions 191–196 of CA I) as crucial for mAb 2B8′s interaction. In 3-D structural analysis, this sequence is spatially adjacent to a previously identified epitope (DFWTYP) that interacts with patient-derived autoantibodies. Critically, mAb 2B8 demonstrated an ability to infiltrate eukaryotic cells, engaging specifically with its intracytoplasmic target. This positions mAb 2B8 as a promising model for future studies aimed at tumor cell eradication.

**Summary**

• Anticarbonic anhydrase I (CA I) autoantibodies from multiple myeloma patients dose-dependently impact CA I esterase activity.
• The newly developed anti-CA I mAb 2B8 exhibits a similar inhibitory effect to patients' autoantibodies.
• The identified linear epitope for anti-CA I mAb 2B8 comprises the amino acid sequence DFWTYP.
• DFWTYP, the mAb 2B8 epitope, is in close proximity to the spatially adjacent to a previously identified epitope (DFWTYP) epitope recognized by patients' autoantibodies.

## 1. Introduction

Carbonic anhydrases (CAs) form a large family of zinc metalloenzymes that show extensive diversity in their tissue distribution and subcellular localization [1, 2]. Among them, CA I (EC 4.2.1.1) represents a cytosolic isoform that is essential for a in a plethora of biological processes (respiration, calcification, acid–base balance, bone resorption, etc.) [3]. Its abundant occurrence in erythrocytes has further accentuated its relevance, elevating it as a critical yardstick for erythroid differentiation [4]. It catalyzes the reversible hydration of carbon dioxide to bicarbonate anion (HCO_3^−^_) and a proton (H^+^). The reactivity of this enzyme is based on the cooperation of zinc (II) ion and three histidine residues (His 94, His 96, and His 119) [5].

However, CA I's sphere of influence is not restricted to physiological processes alone. It is intricately woven into an array of pathophysiological narratives, ranging from the modus operandi of cancer cell proliferation to the complex tapestry of autoimmune reactions and inflammatory cascades [6–16]. Given its potential association with prevalent cancers, such as breast, colorectal, lung, and prostate, CA I is increasingly recognized for its diagnostic and prognostic value [17–20]. For instance, low CA I levels in colonic epithelial cells have been identified as a specific marker for predicting colorectal cancer [21]. Conversely, elevated CA I levels have been observed in the sera of patients with stage I non-small cell lung cancer [19] and in the plasma of prostate cancer patients compared to healthy controls [18]. Differences in CA I expression have also been found in erythroleukemia [22], intestinal metaplasia [23], and several other types of tumors, including hepatocellular carcinomas, cholangiocellular carcinoma [20], and gastric carcinomas [24].

Moreover, the presence of anti-CA I antibodies in diverse disease landscapes further crystallizes its diagnostic prowess and prognostic potential [9, 25, 26]. Recent insights have drawn attention to the occurrence or increased abundance of anti-CA I antibodies in correlation with the course of the disease or the ability of patients to respond well to treatment [6, 8, 9, 26–28]. For instance, high titers of anti-CA I autoantibodies were observed in sera of patients with Hodgkin's disease, multiple myeloma, and some other hemato-oncological malignancies after high-dose therapy (HDT) combined with autologous stem cell transplantation (ASCT) [29]. A correlative aspect that arose from these observations was the manifestation of AA or AA-like syndrome, hinting at a profound autoimmune response potentially targeting the underlying malignancy. The underlying hypothesis is that pancytopenia, observed in these conditions, might be a reflection of a broader autoimmune cascade that is tactically dismantling the malignancy [30–32].

Diving deeper into this paradigm, this investigation is aimed to assess their impact on the catalytic activity of CA I and seek answers to pivotal queries: Do these antibodies ignite antitumor cascades after fusion with malignant cells, or is their focus largely on CA I molecules which influence the central metabolic trajectory of the cells? Addressing this requires a uniform monoclonal antibody that reflects the reactivity profile of innate polyclonal anti-CA I IgG. While commercially available counterparts did not meet this criterion, our collaboration with Moravian-Biotechnology Ltd. set a new direction. We procured a monoclonal antibody tailored to our specific needs. This effort further focuses on mapping the intersections between our new monoclonal antibody and internal polyclonal anti-CA I IgG using cutting-edge methodologies, including epitope extraction and phage display techniques.

## 2. Materials and Methods

### 2.1. Reagents and Chemicals

CA I from human erythrocytes, acetazolamide, 4-nitrophenyl acetate (4-NPA), acetonitrile, Tris (hydroxymethyl)aminoethane (Tris), Ponceau S, DL-dithiothreitol (DTT), iodoacetamide (IAA), and trypsin treated with L- (tosylamido-2-phenyl) ethyl chloromethyl ketone (TPCK) were purchased from Sigma–Aldrich (St. Louis, MO, USA). BSA was from Promega, Madison, WI, USA, formic acid (FA) from Merck, Darmstadt, Germany, and trifluoroacetic acid (TFA) from Fluka, Buchs, Switzerland. Perloza MT 500 (80–100 µm) was product of Iontosorb (Lovosice, CZ). SiMAG-Carboxyl magnetic particles were from Chemicell, Berlin, Germany, Protein G Sepharose 4 Fast Flow beads from GE Healthcare, Little Chalfont, UK, Protein A Sepharose CL-4B from Amersham Biosciences, Piscataway, NJ, USA, Dynabeads Protein G from Invitrogen, Carlsbad, CA, USA, and Sera-Mag SpeedBeads magnetic carboxylate-modified particles from Thermo Fisher Scientific, Waltham, MA, USA. Control serum (human) I was obtained from BioSystem (Barcelona, Spain). Flebogamma 5% (immunoglobulinum humanum normale, IVIg) was purchased from Instituto Grifols, S.A. (Barcelona, Spain). Clarity western ECL substrate was purchased from Bio-Rad (Hercules, CA, USA). Nitrocellulose membrane was from Schleicher Schuell, Dassel, Germany. Other chemicals were reagent grade suitable for analysis and supplied by Lach-Ner (Neratovice, CZ).

### 2.2. Polyclonal Anti-CA I IgG Molecules

(a) Representative anti-CA I IgG autoantibodies were isolated previously [33, 34] from a positive serum of a hematooncological patient after the HDT and ASCT. The autoantibodies were stored at −80°C until use. (b) Goat polyclonal anti-hu CA I hyperimmune serum, supplied by Fitzgerald (Acton, MA, USA).

### 2.3. Production of Anti-CA I Monoclonal Antibody

The monoclonal anti-CA I IgG antibodies were prepared by standard hybridoma technology based on the immunization of mice with CA I isoform isolated from human erythrocytes in the frame of a contract with Moravian-Biotechnology Ltd. (Brno, Czech Republic).

### 2.4. Determination of IC_50_ of Specific Inhibitor Acetazolamide

The reaction mixture was prepared in a total volume of 150 µl, and it was composed of 5 µl of 2.0 × 10^−5^ M CA I, 5 µl of acetazolamide (in the range from 1 × 10^−3^–1 × 10^−7^ M), 20 µl of redistilled water, 70 µl of 50 mM Tris-SO_4_ buffer pH 7.5 and 50 µl of 3 mM 4-NPA. Incubation ran for over 10 min in the dark at 37°C, supplemented by gentle stirring. The esterase activity of CA I was measured by a spectrophotometer at 405 nm.

### 2.5. Determination of CA I Esterase Activity

The two-step protocol included preincubation (0–180 min) of 5 µl CA I (2.0 × 10^−5^ M corresponding to 2.9 µg of CA I and 0.6 Wilbur–Anderson units; with 5 µl tested sample (antibodies in CA I: IgG molar ratios 1:0.1, 1:0.25, 1:0.5 and 1:1 or acetazolamide in the range 1 × 10^−3^–1 × 10^−7^ M). Subsequently, 20 µl of redistilled water, 70 µl of 50 mM Tris-SO_4_ buffer pH 7.5, and 50 µl of 3 mM substrate 4-NPA [35] were added to the preincubated mixture. Then, it was incubation for 10 min at 37°C in darkness under gentle stirring, and the esterase activity was determined.

### 2.6. Dot-Blot Analysis of Patient's Anti-CA I IgG or Anti-CA I mAb Reactivity

Dot-blot experiment was performed according to Svobodova [36] with slight modifications. Native and unfolded CA I samples of 1 µg were dissolved in 100 µl of 10 mM phosphate buffer pH 7.3 and then spotted on PVDF membrane equilibrated by 100 µl of PBS-T buffer. Then PVDF membrane was blocked by 5% milk powder in PBS-T for 60 min. The 60 min incubation step with patient anti-CA I antibodies was followed. Then, a secondary antibody (anti-human IgG/HRP) diluted in the proper ratio was applied. Finally, washing with PBS-T and chemiluminescence detection by Clarity western ECL substrate were carried out. ChemiDoc XRS + Imaging System with Image Lab Software (Bio-Rad, Hercules, CA, USA) was applied for documentation.

### 2.7. Western Blot Analysis

CA isoforms (I, VA, VB, IX, and XII; Supporting Information 1: Table S1; 1.4 pmol) were dissolved in a solution containing 8% (w/v) sodium dodecyl sulfate (SDS), 20 mM Tris–HCl buffer pH 6.8, 40% (v/v) glycerol, 10 mM ethylenediaminetetraacetic acid and 0.2% bromophenol blue. After Laemmli's electrophoretic separation (PROTEAN 3 from Bio-Rad, Hercules, CA, USA), in 12.5% SDS-PAGE gel, the proteins were transferred onto nitrocellulose membrane by semi-dry blotting apparatus (Bio-Rad, Hercules, CA, USA). The quality of protein transfer was controlled by staining with 0.1% Ponceau S in 5% acetic acid. The membrane blocked with 1% blot quantified BSA was incubated overnight with mAbs (10^3^, 10^4^, and 10^6^ dilutions; stock concentration 1 mg/ml). After washing steps, the membrane was incubated with anti-mouse IgG (H&L) alkaline phosphatase conjugate (Promega, Madison, WI, USA) (10^6^ dilutions) and detected with 5-bromo-4-chloro-3-indolyl phosphate/nitro blue tetrazolium reagent (Promega, Madison, WI, USA).

### 2.8. Immunofluorescence Assay

PC3 and VERO cells (30,000 cells) were seeded onto a 3.5 cm culture dish containing sterile coverslips and incubated overnight (humidity 37°C, 5% CO_2_). After washing with PBS (pH 7.2), the cells were fixed with ice-cold methanol for 5 min and washed again (PBS). Unspecific binding of the antibodies was blocked by 1% BSA in PBS (pH 7.2) for 30 min at 37°C. Then, the cells were incubated with FITC labeled (Exbio, Czech Republic) anti-CA I mAb in a humidified chamber for 1 h at 37°C, followed by washing the coverslips in PBS. Finally, one drop (5–6 µl) of the Vectashield mounting medium for fluorescence with DAPI (Vector laboratories, USA) was added to each coverslip and covered with another slide. The slide edges were then coated with nail polish and stored in the dark at 4°C. The cells were scanned on scanning confocal laser scanning microscope LSM 510 META (Zeiss, Germany) using a 40x oil immersion objective.

### 2.9. Unfolding and Proteolytic Digestion of CA I Molecules

CA I from human erythrocytes (1 mg; Sigma–Aldrich, St. Louis, MO, USA) was unfolded by 0.1% RapiGest SF (Waters, Milford, MA, USA) combined with reductive alkylation using 50 mM DTT and 100 mM IAA in 50 mM ammonium bicarbonate solution. TPCK trypsin (13,816 units/mg) immobilized on SiMAG-Carboxyl magnetic particles (1 µm) was applied for digestion in enzyme: substrate molar ratio 1:20 for 2.5 h at room temperature (RT) under mild stirring. The one-step carbodiimide method was used for TPCK-trypsin immobilization [37].

### 2.10. Immobilization of Anti-CA I IgG to Magnetic Microparticles

The patients' anti-CA I IgG or anti-CA I mAb (100 µg) were covalently attached to 2 mg of Sera-Mag SpeedBeads magnetic particles (0.816 µm) by two-step carbodiimide method consisting of preactivation of the beads with 80 mM 1-ethyl-3- (3-dimethylaminopropyl)-carbodiimide hydrochloride and 10 mM N-hydroxysulfosuccinimide sodium salt, followed by immobilization of the IgG molecules in 100 mM 2-(N-morpholino)ethanesulfonic acid buffer pH 5.0 overnight at 4°C and under mild stirring.

### 2.11. Epitope Extraction: Immunoprecipitation of the CA I or Its Tryptic Fragments

The native unfolded (20 µg) or digested (40 µg) CA I in 0.5 ml of phosphate buffer pH 7.0 were added to magnetic beads-based immunosorbent (Sera-Mag microparticles, 0.5 mg) biofunctionalized by patients' anti-CA I IgG or anti-CA I mAb, prewashed with the same buffer, and incubated for 1.5/2.5 h at RT under mild stirring. Then, the microparticles were intensively washed in three consecutive steps: with phosphate buffer pH 7.0 (containing 0.2 and 1 M NaCl), 10x diluted phosphate buffer pH 7.0, and ultra-pure water. Finally, the elution of immunocaptured CA I molecules or its peptide fragments was performed by 0.05% (v/v) TFA treatment at RT for 15 min under mild stirring into three fractions (3 × 0.2 ml) [33, 34, 38]. The eluates were dried in speed-vac and analyzed by SDS–PAGE (10% [w/v] separating gel) or mass spectrometry (MS).

### 2.12. Mass Spectrometric Analysis

PC3 cells (10^6^) were incubated for 2 h with 100 µg/ml of mAb 2B8, washed in PBS, and resuspended in 0.1% 2-mercaptoethanol, 1% protease inhibitor, and 1% EDTA. The sample was homogenized by pathing through a needle, and the protein was extracted using the chloroform-methanol method. Proteins were reduced with 10 mM DTT at 50°C, alkylated with 50 mM IAA, and washed with ammonium bicarbonate. Trypsin digestion (2 µg in 75 µl ammonium bicarbonate) was carried out overnight at 37°C.

Peptides collected from PC3 cells, as well as the captured CA I-specific peptide fragments from epitope extraction, were analyzed by an automated nanoflow UPLC system coupled to a Q-TOF Premier (Waters, Milford, MA, USA) tandem mass spectrometer (LC–MS/MS). Peptides injected onto a reverse-phase column (nanoAcquity UPLC column BEH 130 C18, 75 μm × 150 mm, 1.7 μm particle size) were separated using the acetonitrile gradient (3%–40%) with 0.1% FA in 15 min at a flow rate of 350 nl/min. The column was directly connected to the PicoTip emitter (New Objective, Woburn, MA, USA) and mounted into the nanospray source. For protein identification, a multiplex approach called MSE was used [39]. The obtained MS/MS data were processed using the ProteinLynx Global Server v. 2.4 (Waters, UK). The resulting data were searched against Swissprot and the specific CA I sequence databases using previously described parameters [33]. The protein groups were analyzed for differential expression by applying a cut-off value for the ANOVA test of *p*  < 0.05.

### 2.13. Phage Display Epitope Mapping

Briefly, 3 µl of 10^9^ phage-containing 12-mer peptides (Ph.D.-12 phage-display library, New England Biolabs, Ipswich, MA, USA) were incubated for 2 h at 4°C under mild stirring with 50 µl of ~20 µg selected mAb attached to Protein G Sepharose in PBS containing 0.1% BSA. Antibody-bound phage was eluted in 100 µl of 0.1 M glycine buffer pH 2.5 and neutralized by 0.1 M Tris pH 8.5. The selected phage was amplified in *Escherichia coli* (host strain ER2738) and taken through an additional two cycles with anti-CA I antibody attached to different beads: Protein A Sepharose CL-4B and Dynabeads Protein G. After three rounds, the selected phage was placed with *E. coli* (host strain ER2738) on lysogeny broth (LB)/isopropyl-*β*-D-thiogalactoside (IPTG)/5-bromo-4-chloro-3-indoyl-*β*-D-galactoside (Xgal) agar plate and incubated at 37°C overnight. Positive plaques were consequently picked for PCR amplification of phage DNA. The DNA containing the variable region was amplified by Herculase II Fusion DNA Polymerase (Agilent Technologies, Santa Clara, CA, USA) using forward primer 5′cgtgggcgatggttgttgtc3′ and reverse primer 5′taagtgccgtcgagagggttgata3′. The PCR product was purified using a QIAquick PCR purification kit (QIAGEN Inc., Valencia, CA, USA). Finally, the DNA was sequenced using primer 96GIII 5′ccctcatagttagcgtaacg3′. The sequences coding the variable region of 12 amino acids were translated to peptides. The peptide sequences were analyzed by multiple alignments using the T-Coffee algorithm. Sequence logo generator WebLogo visualized the consensus sequence of the epitope.

## 3. Results

### 3.1. Analyzing Anti-CA I Autoantibody Reactivity

The foundation of our analysis rested upon a direct method of estimating CA I activity, which involved the conversion of the substrate 4-NPA to 4-nitrophenolate. During preliminary tests, we ascertained a consistent linear signal within the range of 1.0 × 10^−5^–4.0 × 10^−5^ M for CA I molar values. This led us to anchor our subsequent experiments around a concentration of 2.0 × 10^−5^ M of CA I.

We chose acetazolamide, belonging to the sulfonamides group, as the representative inhibitor specific to *α*-CAs. This compound reacts with amino acids present within CA I's active site, thereby establishing a durable complex [1, 40, 41]. Initial analyses estimated an effective inhibitor concentration of 5.8 × 10^−5^ M for a 50% reduction in CA I activity (Supporting Information 2: Figure S1).

**Figure 1:**
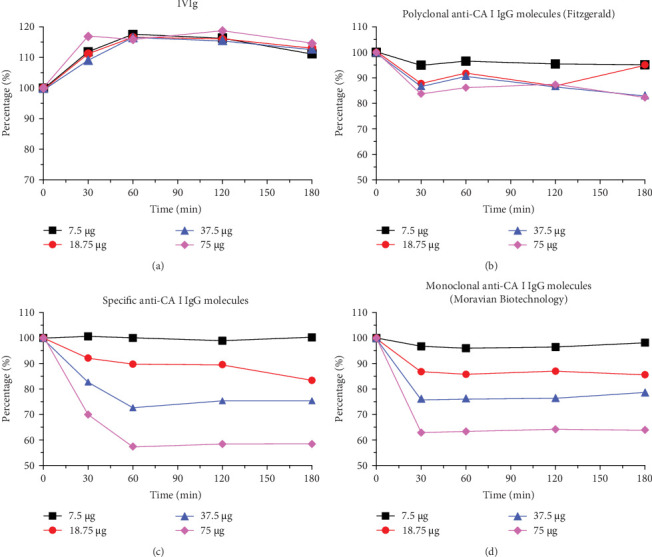
Inhibition effect of anti-CA I IgGs to CA I enzyme activity (2.0 × 10^−5^ M, 15 µg, reaction volume 50 µl). (A) 7.5–75 µg IgGs collected from healthy donors (IVIg), (B) 7.5–75 µg goat polyclonal anti-CA I IgG (Fitzgerald, MA, USA), (C) 7.5 – 75 µg anti-CA I IgG isolated from highly positive serum, and (D) 7.5–75 µg mouse monoclonal anti-CA I IgG 2B8 (Moravian Biotechnology). Anti-CA I, antibodies against CA I; IVIg, immunoglobulinum humanum normale.

Our next focal point was gauging the enzymatic activity of CA I when subjected to anti-CA I autoantibodies (IgG). It is pivotal to highlight that these autoantibodies were not freshly isolated; instead, they were drawn from our earlier studies [33, 34]. These antibodies were initially sourced from a multiple myeloma patient serum who was in prolonged remission. The antibodies had been stored at −80°C after being isolated using a covalent-bound CA I column. We utilized 15 µg of CA I and coincubated it with varying amounts of anti-CA I IgG to achieve molar ratios of 1:0.1, 1:0.25, 1:0.5, and 1:1, corresponding to 7.5, 18.75, 37.5, and 75 µg of the antibody, respectively. Over a period of 180 min, samples were taken at 30-min intervals to assess the enzymatic activity. The result was then contrasted to the inhibitory effect of acetazolamide and a commercial polyclonal anti-CA I IgGs, as well as to IgG collected from healthy donors (IVIg) as a negative control (Figure 1). While the IVIg fraction remained unaltered (Figure 1A), polyclonal anti-CA I IgG from goats registered only a modest activity decrease (up to 20%) (Figure 1B). Yet, the preserved natural anti-CA I IgG showcased a pronounced dose-dependent impact, mirroring the inhibition capabilities of acetazolamide at a 1:1 molar ratio (Figure 1C).

### 3.2. Monoclonal Antibody Development and In Vitro Reactivity

To deepen our understanding of the implications of anti-CA I autoantibodies in antitumor immunity, it was pivotal to obtain IgG molecules exhibiting consistent reactivity akin to natural anti-CA I IgG. In collaboration with Moravian-Biotechnology Ltd. in Brno, Czech Republic, we successfully produced distinct anti-CA I monoclonal antibodies. They were prepared by standard hybridoma technology [42, 43]. The CA I antigen sourced from human erythrocytes was introduced to immunize mice. A subsequent western blot analysis of nine hybridoma cell clones revealed their binding capabilities with the target antigen. Notably, antibodies from the clone 2B8 (Supporting Information 3: Figure S2, position 1) displayed the most promising reactivity with CA I and were thus selected for further experimental procedures.

The next assessment was directed toward the cross-reactivity of the developed mAb 2B8 with other CA isoforms. This was vital given that cross-reactivity is a significant bottleneck when aiming for the application of specific anti-CA I antibodies. Our examination spanned across five major CA isoforms: cytosolic CA I, mitochondrial CA VA and CA VB, and membrane-bound CA IX and CA XII. Western blot outcomes, as illustrated in Supporting Information 3: Figure S2, accentuated mAb 2B8′s distinctive specificity to isoform CA I (position 1). The mAb displayed zero reactivity with isoforms CA IX and CA XII (positions 4 and 5). However, faint signals were noted for CA VA and CA VB isoforms (positions 2 and 3).

Further, we embarked on evaluating the inhibitory prowess of this antibody. Specific quantities (7.5, 18.75, 37.5, and 75 µg) of the anti-CA I IgG were incubated with 15 µg of CA I in a final volume of 50 µl over a span of 180 min. An intriguing observation was that the monoclonal antibody manifested a 50% inhibitory impact in a mere 30 min (Figure 1D). This was considerably faster compared to the patient-derived IgGs (Figure 1A). Such an observation propounds that the anti-CA I mAb 2B8 is poised to serve as an exemplary model antibody for both *in vitro* and *in vivo* assays. There is a tangible potential to empirically validate its capability to instigate cellular damage culminating in tumor cell death, drawing parallels with previous findings [44, 45].

Subsequently, we employed dot blot analysis to delve deeper into the binding preference of mAb 2B8. Specifically, we sought to determine its affinity towards native protein structures in comparison to their unfolded or digested versions. Supporting Information 4: Figure S3 presented a compelling visualization: mAb 2B8 exhibited robust immunoreactivity across all tested antigenic forms. Most notably, heightened reactivity was discerned with unfolded proteins (position 2) and tryptic digests of CA I (position 3). Given that alterations in the native conformation of CA I (position 1) did not diminish the reactivity of the anti-CA I IgG, we postulate that the epitope of CA I that mAb 2B8 targets is predominantly linear rather than being conformational.

To further authenticate these findings, we then anchored mAb 2B8 to magnetic beads via a covalent bond, aiming to observe its reactivity with an unfolded form of CA I. Following the capturing process, CA I (unfolded state) was delicately eluted and subsequently subjected to SDS–PAGE analysis. As depicted in Supporting Information 5: Figure S4, the immobilized 2B8 antibody adeptly captured unfolded CA I (positions 7–9) and position 10 verify the full elution of captured CA I in prior fractions. A pronounced reduction in protein concentration (~80%) was conspicuous for the unfolded CA I (position 2) post a 1.5-h incubation with the immunosorbent. These results not only echo but also reinforce the observations derived from our dot blot assessment.

### 3.3. Investigating Antibody–Antigen Interactions in Native States

The efficacy of anti-CA I antibodies in tumor elimination is not just contingent on their mere presence but also on their inherent capability to traverse cellular membranes. Such a dual characteristic plays a pivotal role in eradicating tumor cells, as evidenced in several studies [1, 46–50].

**Figure 2:**
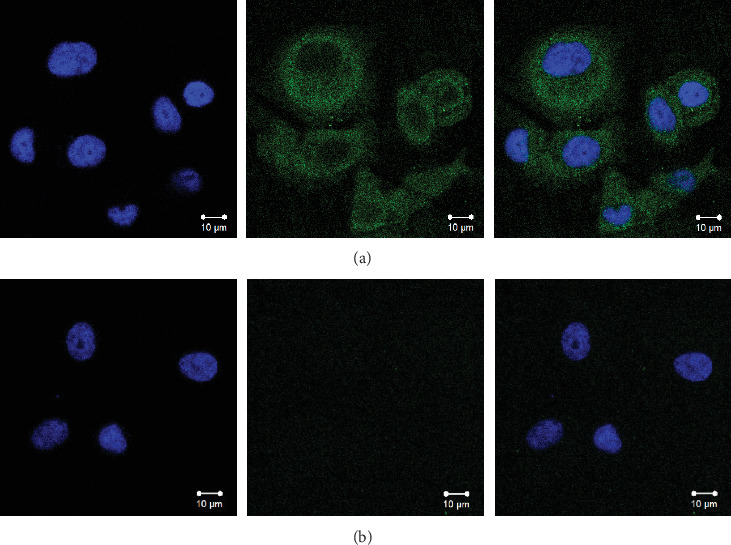
Confocal microscopy of PC3 and VERO cells incubated with the mAb 2B8. Cell nuclei were stained with DAPI (blue signal) and the anti-CA I mAb 2B8 with FITC (green signal). (A) PC3 cells and (B) VERO cells. Anti-CA I, antibodies against CA I.

To empirically ascertain whether our newly synthesized anti-CA I mAb 2B8 possesses the capability to traverse cellular membranes, we embarked on a detailed investigation. We cultivated two distinct cell lines: PC3, known to be CA I positive, and VERO, a CA I negative counterpart. Both cell lines were exposed to mAb 2B8, with the interactions meticulously captured on microscopic slides (Figure 2). Intriguingly, confocal microscopy revealed a compelling narrative. The monoclonal anti-CA I antibody did not just permeate the PC3 cell membrane but also demonstrated precise binding to CA I within the cytoplasm, as illustrated in Figure 2A. In stark contrast, the VERO cells, being CA I negative, showed no discernible interaction with the antibody (Figure 2B).

Moreover, an initial proteomic analysis of PC3 cells treated with mAb 2B8 revealed notable changes in the proteomic repertoire, further supporting the antibody's influence on cellular processes. Principal component analysis (PCA) of these cells demonstrated clear clustering within each group (treated and control), with slight overlap, indicating that while many proteins remained consistent, there were distinct differentially regulated proteins in the mAb-treated group (Supporting Information 6: Figure S5). Although further proteomic analyses are needed to confirm these findings, this initial observation suggests that mAb 2B8 may induce specific proteomic alterations that could contribute to its antitumor mechanisms.

### 3.4. Epitope Identification Techniques

Finally, we decided to accurately identify the primary immunogenic epitope of CA I that interacts with mAb 2B8. Initiating our approach with the epitope extraction technique, CA I underwent digestion with immobilized TPCK-trypsin. Postdigestion, the fragmentation's efficiency was confirmed through LC–MS/MS analysis (Supporting Information 7: Figure S6 A). We then introduced the resultant mixture of CA I tryptic fragments to the immunosorbent preloaded with immobilized anti-CA I IgG. A 1.5-h incubation period ensued, after which we executed a series of washing steps. Eluting the captured peptides and subjecting them to LC–MS/MS analysis, a standout peptide emerged in the third elution fraction, indicative of the strongest interaction affinity. Detailed analysis of this peptide's MS/MS spectrum revealed it corresponded to the sequence: APFTNFDPSTLLPSSLDFWTYPGSLTHPPLYESVTWIICKESISVSSEQLAQFR—a tryptic peptide of CA I with a singular missed cleavage, located at position 175–228 with a theoretical mass of 6160.0364 *m*/*z* (Supporting Information 6: Figure S6 B).

**Figure 3:**
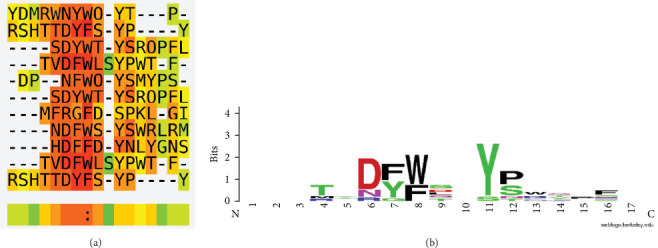
Amino acid sequences identified by phage display peptide library. (A) multiple alignments performed by the T-Coffee algorithm and (B) a consensus of sequences using sequence logo generator WebLogo.

To solidify our findings, we employed the Ph.D.-12 Phage Display Peptide Library, renowned for its combinatorial library of dodecapeptides amalgamated with M13 phage's minor coat protein, pIII. With a vast reservoir of about 10^9^ electroporated sequences, each cycle of amplification yields roughly 10^2^ copies of every sequence in a 10 µl phage sample. Upon incubating this library with beads coated with the target antibody, mAb 2B8, unattached peptides were washed off. The adherent ones were then eluted, amplified, and subjected to successive rounds of binding and amplification, enhancing the pool's specificity. After three such enriching rounds, DNA sequencing of individual clones highlighted 11 peptide sequences interacting with the antibody (Figure 3A).

**Figure 4:**
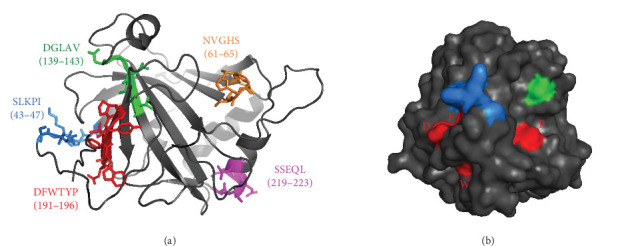
3-D structure of CA I (PDB: 1AZM) depicting the peptide sequence identified by phage display as an epitope for anti-CA I mAb 2B8 together with previously described epitopes of patient autoantibodies [39, 40]. (A) the sequence DFWTYP recognized by mAb 2B8 is highlighted in red, the epitopes of patient autoantibodies NVGHS in ochroid, DGLAV in green, SSEQL in magenta, and SLKPI in blue and (B) the surface localized amino acids are shown—epitope DFWTYP in red, the epitopes recognized by patient's autoantibodies DGLAV in green, and SLKPI in blue. Anti-CA I, antibodies against CA I; DFWTYP, spatially adjacent to a previously identified epitope.

A juxtaposition of the sequence from our initial epitope extraction method with those discerned from phage display pinpointed the sequence DFWTYP, representing amino acids 191–196 of CA I, as a dominant epitope reacting with mAb 2B8 (Figure 3B). Further insights from a 3-D structure analysis showcased that DFWTYP is intrinsic to the *β*-sheet structural framework within the core realm of the native protein (Figure 4A) and is somewhat receptive to antibody binding (Figure 4B). Intriguingly, even though the DFWTYP epitope does not match previously identified epitopes from patients' autoantibodies [33, 34], it bears a striking spatial proximity to the spatially adjacent to a previously identified epitope (DFWTYP) sequence in the 3-D structural landscape (Figure 4). This proximity leads us to speculate that the newly synthesized mAb 2B8 and the autoantibodies from patients likely target a shared protein region, exerting similar effects.

## 4. Discussion

This study builds upon our previous foundational works, wherein we observed anti-CA I autoantibodies in sera of patients with hemato-oncological malignancies [44] and identified four primary immunodominant epitopes: NVGHS, DGLAV, SSEQL, and SLKPI displaying affinity for anti-CA autoantibodies derived from patients with multiple myeloma who spontaneously regressed after HDT and ASCT [33]. These patients exhibited AA-like syndrome symptoms, suggesting a complex interplay between their therapeutic regimen and immune responses [30, 31]. The same epitopes were also identified in patients with bona fide AA, showcasing varied severity degrees [34]. Studying the three-dimensional structure of native CA I illuminated the differential surface exposure of these epitopes, which can considerably influence their accessibility to autoantibodies [33, 34].

Delving into the intricate dynamics between anti-CA I autoantibodies and tumor cell behavior, we further focused on the influence of patients' anti-CA I positive sera on the in vitro growth of PC3 and MDA-MB-231 cells. Our observations revealed marked shifts in mRNA expression profiles for various proteins, notably those involved in the extracellular matrix. During the treatment regimen, there was an uptick in the CA1 gene expression across both cell lines. Concurrently, there was a noticeable downregulation in the expression of genes responsible for coding collagen type I alpha 1, collagen type IV alpha 4, laminin subunit gamma 2, collagen triple helix repeat containing 1 gene, and the Wnt family member 7B. These molecular changes were accompanied by discernible morphological alterations in the in vitro cultured tumor cells. Interestingly, despite these molecular changes, the presence of anti-CA I autoantibodies did not impair tumor cell proliferation and cell viability in vitro [51]. On the other hand, when CA I expression was silenced via mRNA knock-down, the fallout was a surge in mRNAs coding for collagen I and IV across both cell lines [52].

These observations paralleled with a broader notion—the potential of anti-CA I antibodies to inflict cellular damage and contribute to the elimination of tumor cells. This damage is believed to stem from their inhibitory impact on the catalytic activities of their target proteins [25, 50]. Herein, we assessed the reactivity of patients' anti-CA I autoantibodies with the target molecules. We showed that the natural anti-CA I IgG has a dose-dependent impact, mirroring the inhibition capabilities of acetazolamide at a 1:1. Delving into this inhibitory potential, Botre et al. [25] highlighted the significant impact of both anti-CA I and anti-CA II polyclonal antibodies on enzymatic functionality. In their unique context, they sourced antibodies from patients with primary biliary cirrhosis and posited that these antibodies, given their ability to impede CA's catalytic function, might contribute to the degradation of biliary epithelial cells. This hypothesis was further reinforced by Adamus and Karren [53], who explored the antibodies' pathogenic role in autoimmune and paraneoplastic retinopathies.

Positioning our current study within this intricate tapestry, we underscore the nuanced interplay between autoimmunity, enzymatic functions, and tumor dynamics. To address this, we sought a consistent monoclonal antibody resembling the properties of naturally occurring polyclonal anti-CA I IgG. In collaboration with Moravian-Biotechnology Ltd., we procured hybridoma cells yielding specific anti-CA I monoclonal antibodies. Subsequent verification was conducted using advanced techniques to compare these antibodies with human polyclonal anti-CA I IgG. The synthesized mAb 2B8, our bespoke mouse monoclonal IgG antibody, exhibited comparable efficacy. Deeming mAb 2B8 as a potential reference for both in vitro and in vivo assays, our structural assessment unveiled that the DFWTYP epitope, inherent to the native protein's *β*-sheet domain, bears a significant spatial congruence with the previously identified SLKPI sequence. This hints at the potential of mAb 2B8 and patient-derived autoantibodies converging on a mutual protein region, culminating in analogous outcomes. Initial proteomic analysis of mAb 2B8-treated PC3 cells further supported these findings by revealing changes in protein expression profiles, suggesting its broader influence on cellular pathways.

Yet, the enigma of spontaneous tumor regression posttreatment remains. Though extensively documented in conditions like lymphomas, leukemias, melanomas, neuroblastomas, and renal cell carcinomas [54–58], the underlying mechanics are still under scrutiny. While theories abound, spanning immune mediation to hormonal interactions, our revelations offer a fresh vantage point. The pronounced presence of anti-CA I autoantibodies during spontaneous tumor regression, paralleled by bone marrow suppression observed as an “AA-like syndrome,” demands further inquiry. The observed marrow suppression in conditions such as SLE, coupled with corresponding autoantibodies, accentuates the prospective cross-reactivity of these autoantibodies against other pivotal targets, possibly including the DNA polymerase theta [30, 31].

## 5. Conclusion

Our in-depth exploration illuminates the intricate relationship between anti-CA I autoantibodies, found in patients experiencing spontaneous tumor regression following HDT and ASCT, and CA I's esterase function. These patients notably mirrored varying severities akin to aplastic anemia-like syndrome. Our findings spotlight the formidable capacity of these autoantibodies to modulate CA I's esterase activity in a dose-dependent manner. In circumstances where the concentration of the autoantibodies is equivalent to the enzyme's molar ratio (1:1), their inhibition potency rivals that of the well-established inhibitor, acetazolamide, as reflected in their congruent IC_50_ values.

This observation is harmoniously echoed in our newly developed mAb 2B8. To discern any potential similarity between these IgGs, their binding epitopes were analyzed side by side. For this endeavor, we embraced a comprehensive dual-pronged strategy, integrating epitope extraction with the phage display peptide library methodology. Through this lens, the amino acid sequence DFWTYP, spanning positions 191–196, stood out as crucial for mAb 2B8′s binding capacity. Fascinatingly, while DFWTYP is linear, its 3-D spatial conformation exhibits a close affinity with the earlier identified SLKPI sequence, which is a known target for anti-CA I autoantibodies derived from patients.

Bolstered by the premise that the presence of anti-CA I autoantibodies in serum potentially enhances patient survival outcomes, our study suggests that mAb 2B8 is well-poised to serve as an exemplar model. It offers a fresh perspective into the intricate processes steering tumor cell eradication. Beyond its scholarly relevance, the emergence of mAb 2B8 also paves the way for pioneering therapeutic interventions, potentially ushering in a transformative era for patients contending with tumors.

## Data Availability

The data that support the findings of this study are available from the corresponding author upon reasonable request.

## Supporting Information

Supporting Information 1 Table S1: List of carbonic anhydrase (CA) isoforms used for evaluation of their immunoreactivity with the 2B8 monoclonal antibody by western blot analysis.

Supporting Information 2 Figure S1: Inhibitory effect of acetazolamide on carbonic anhydrase I (2.0 × 10^−5^ M) via esterase activity monitoring. (A) Determination of IC_50_: Five different concentrations of acetazolamide (ranging from 10^−3^ M to 1 × 10^−7^ M were tested and (B) time-dependent inhibitory effect of acetazolamide at IC50 (5.8 × 10^−5^ M).

Supporting Information 3 Figure S2: Western blot analysis showing the immunoreactivity of (A) CA I isoform (1.4 pmol) with all developed monoclonal antibody (mAb) clones (crude hybridoma supernatants)—lanes: (1) 1A6 mAb, (2) 2A5 mAb, (3) 2B8 mAb, (4) 6B10 mAb, (5) 7B8 mAb, (6) 2D10 mAb, (7) 6D9 mAb, (8) 2G9 mAb, (9) 7H10 mAb, (10) whole mouse serum, dilution: (1−9) 5x, (10) 200x and (B) CA isoforms (1.4 pmol) with purified 2B8 monoclonal antibody (stock solution 1 mg/ml), dilution of 2B8 antibody 100,000x—lanes: (1) CA I, (2) CA II, (3) CA VA, (4) CA VB, (5) CA IX, (6) CA XII.

Supporting Information 4 Figure S3: Dot-blot analysis: immunoreactivity of mAb 2B8 with various forms of CA I: (1) native CA I (5 µg), (2) CA I unfolded by RapiGest and treated with DTT and IAA (5 µg), (3) unfolded CA I digested by TPCK-trypsin (5 µg), (4) BSA (5 µg), and (5) 10 mM phosphate buffer pH 7.0; dilution: 1000x; dilution of secondary HRP-conjugated antibody: 1000x; colorimetric detection: Opti-4CN kit.

Supporting Information 5 Figure S4: Coomassie brilliant blue stained SDS–PAGE (10% gel) analysis following immunoprecipitation of (A) native and (B) unfolded CA I (20 µg) using magnetic immunosorbent SeraMag (2 mg) coupled with mAb anti-CA I IgG, clone 2B8 (100 µg)—lanes: (1) molecular markers (Bio-Rad, Hercules, CA, USA), (2) initial fraction of native/unfolded CA I, (3) fraction of CA I after incubation (1.5 h) with immunosorbent, (4) first washing fraction, (5) first elution fraction, (6) second elution fraction, and (7) third elution fraction.

Supporting Information 6 Figure S5: Initial proteomic analysis of PC3 cells (106) incubated for 2 h with 100 µg/ml of mAb 2B8. (A) Principal component analysis (PCA) illustrating the clustering of PC3 cell samples based on proteomic profiles and (B) Venn diagram displaying the number of proteins uniquely identified in PC3 treated cells, the untreated control, and the number of shared proteins.

Supporting Information 7 Figure S6: The coverage map of CA I showing (A) tryptic peptide fragments detected in the initial sample (73.1% sequence coverage) and (B) tryptic peptide fragments detected in the third elution fraction obtained during the epitope extraction experiment.
