# Supplementary material for: Inhibitory Effect of Human Anti-CA I Autoantibodies and Development of Monoclonal Antibody mAb 2B8 Targeting Carbonic Anhydrase I
Source: Mediators Inflamm. 2024 Dec 30;2024:9981131. doi: 10.1155/mi/9981131 (PMC11703592; doi:10.1155/mi/9981131)
Supplement: Supporting Information 1 — Table S1: List of carbonic anhydrase (CA) isoforms used for evaluation of their immunoreactivity with the 2B8 monoclonal antibody by western blot analysis. [file 9981131.f1.docx]

***Supplementary Table 1.*** *List of carbonic anhydrase (CA) isoforms used for evaluation of their immunoreactivity with the 2B8 monoclonal antibody by western blot analysis.*

| **Isoform** | **Length (aa)^*^** | **Mass [Da]^*^** | **Location (cell)** | **Location (tissue)** | **Tested protein sample** |  |
| --- | --- | --- | --- | --- | --- | --- |
| CA I | 261 | 28,870 | cytosol | [erythrocytes](http://en.wikipedia.org/wiki/Red_blood_cell), [gastrointestinal tract](http://en.wikipedia.org/wiki/Gastrointestinal_tract) | isolated from human erythrocytes (Sigma-Aldrich) | |
| CA II | 260 | 29,246 | cytosol | almost ubiquitous | recombinant (R&D Systems) | |
| CA VA | 305 | 34,750 | mitochondria | liver | recombinant (R&D Systems) | |
| CA VB | 317 | 36,434 | mitochondria | heart, pancreas, kidney, placenta, lung, and skeletal muscle | recombinant (R&D Systems) | |
| CA IX | 459 | 49,698 | [cell membrane](http://en.wikipedia.org/wiki/Cell_membrane) | epithelial cells of gastric mucosa, carcinoma cells | recombinant (R&D Systems) | |
| CA XII | 354 | 39,451 | [cell membrane](http://en.wikipedia.org/wiki/Cell_membrane) | colon, kidney, prostate, intestine, pancreas, ovary, testis, activated lymphocytes, renal cell cancers | recombinant (R&D Systems) | |

***^*^****according to UniProt database; aa – number of amino acids*
