# Supplementary material for: Inhibitory Effect of Human Anti-CA I Autoantibodies and Development of Monoclonal Antibody mAb 2B8 Targeting Carbonic Anhydrase I
Source: Mediators Inflamm. 2024 Dec 30;2024:9981131. doi: 10.1155/mi/9981131 (PMC11703592; doi:10.1155/mi/9981131)
Supplement: Supporting Information 2 — Figure S1: Inhibitory effect of acetazolamide on carbonic anhydrase I (2.0 × 10^−5^ M) via esterase activity monitoring. (A) Determination of IC50: Five different concentrations of acetazolamide (ranging from 10−3 M to 1 × 10^−7^ M were tested and (B) time-dependent inhibitory effect of acetazolamide at IC50 (5.8 × 10^−5^ M). [file 9981131.f2.pptx]

## Slide 1
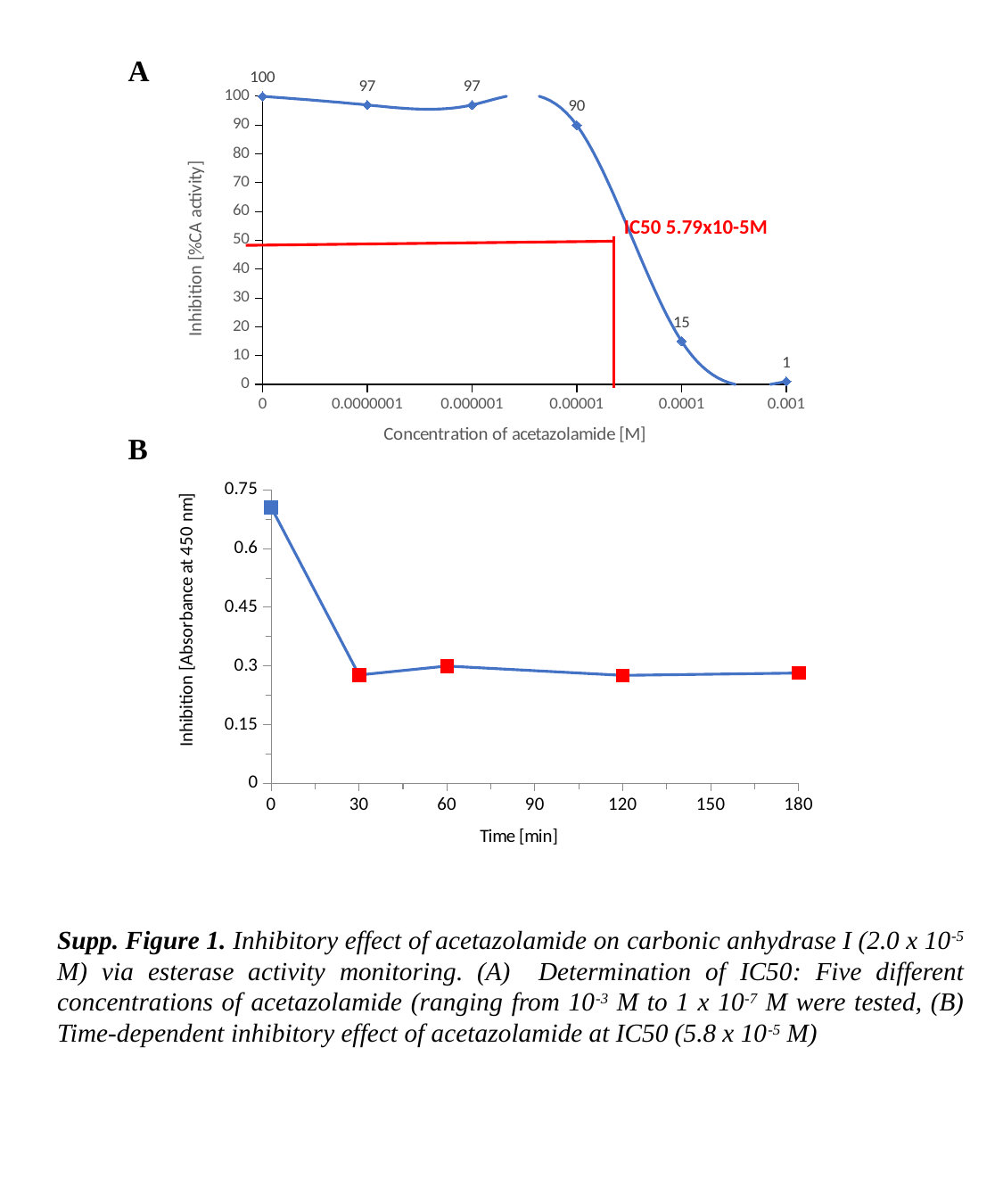

A
### Chart
| Category | |
|---|---|
| 0 | 100.0 |
| 9.9999999999999995E-8 | 97.0 |
| 9.9999999999999995E-7 | 97.0 |
| 1.0000000000000001E-5 | 90.0 |
| 1E-4 | 15.0 |
| 1E-3 | 1.0 |IC50 5.79x10^-5^M
B
### Chart
| Category | |
|---|---|Supp. Figure 1. Inhibitory effect of acetazolamide on carbonic anhydrase I (2.0 x 10^-5^ M) via esterase activity monitoring. (A) Determination of IC50: Five different concentrations of acetazolamide (ranging from 10-3 M to 1 x 10^-7^ M were tested, (B) Time-dependent inhibitory effect of acetazolamide at IC50 (5.8 x 10^-5^ M)
