# Supplementary material for: Inhibitory Effect of Human Anti-CA I Autoantibodies and Development of Monoclonal Antibody mAb 2B8 Targeting Carbonic Anhydrase I
Source: Mediators Inflamm. 2024 Dec 30;2024:9981131. doi: 10.1155/mi/9981131 (PMC11703592; doi:10.1155/mi/9981131)
Supplement: Supporting Information 3 — Figure S2: Western blot analysis showing the immunoreactivity of (A) CA I isoform (1.4 pmol) with all developed monoclonal antibody (mAb) clones (crude hybridoma supernatants)—lanes: (1) 1A6 mAb, (2) 2A5 mAb, (3) 2B8 mAb, (4) 6B10 mAb, (5) 7B8 mAb, (6) 2D10 mAb, (7) 6D9 mAb, (8) 2G9 mAb, (9) 7H10 mAb, (10) whole mouse serum, dilution: (1−9) 5x, (10) 200x and (B) CA isoforms (1.4 pmol) with purified 2B8 monoclonal antibody (stock solution 1 mg/ml), dilution of 2B8 antibody 100,000x—lanes: (1) CA I, (2) CA II, (3) CA VA, (4) CA VB, (5) CA IX, (6) CA XII. [file 9981131.f3.pptx]

## Slide 1
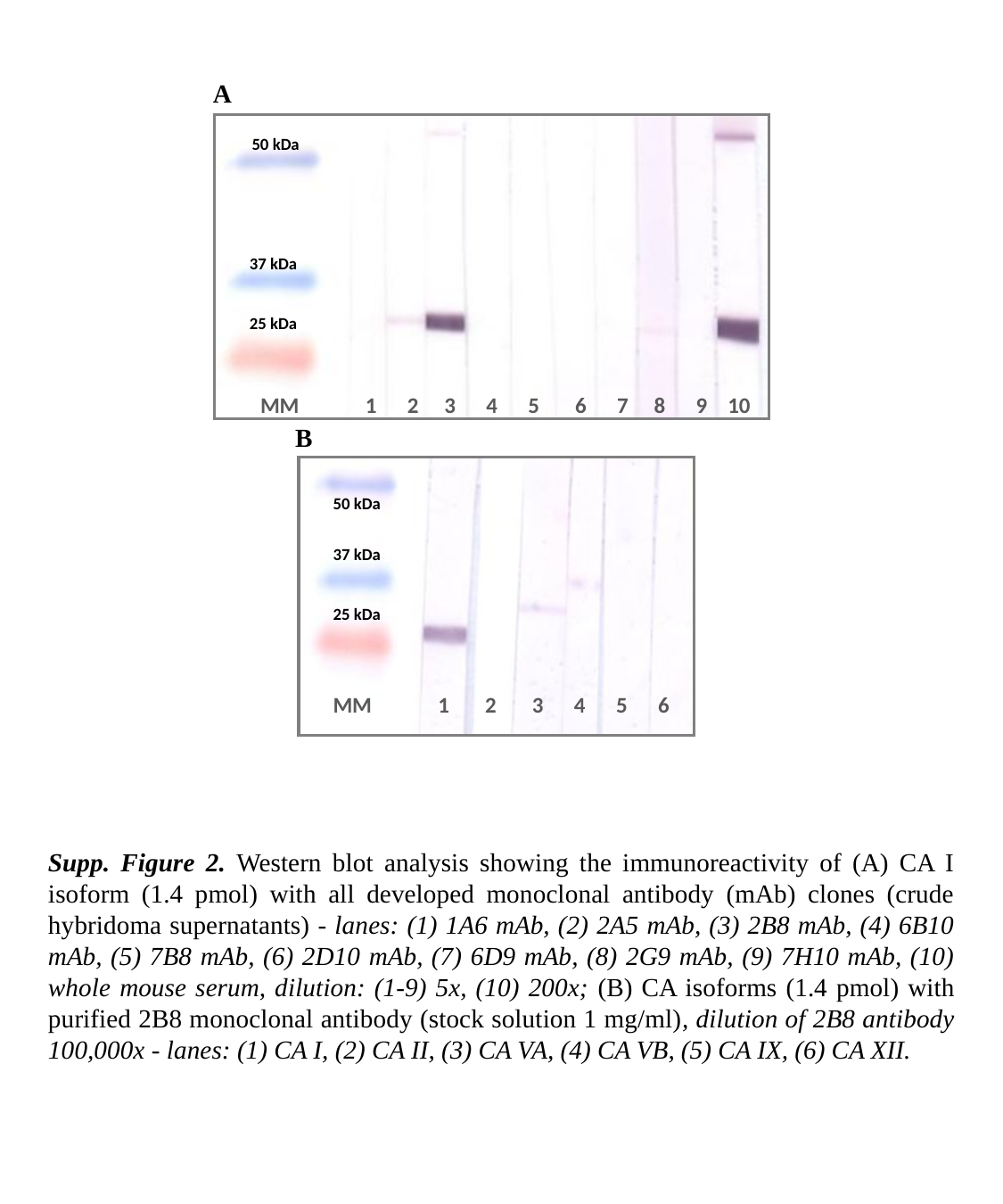

A
 50 kDa
 37 kDa
 25 kDa
 MM 1 2 3 4 5 6 7 8 9 10
 B
 50 kDa
 37 kDa
 25 kDa
 MM 1 2 3 4 5 6
Supp. Figure 2. Western blot analysis showing the immunoreactivity of (A) CA I isoform (1.4 pmol) with all developed monoclonal antibody (mAb) clones (crude hybridoma supernatants) - lanes: (1) 1A6 mAb, (2) 2A5 mAb, (3) 2B8 mAb, (4) 6B10 mAb, (5) 7B8 mAb, (6) 2D10 mAb, (7) 6D9 mAb, (8) 2G9 mAb, (9) 7H10 mAb, (10) whole mouse serum, dilution: (1-9) 5x, (10) 200x; (B) CA isoforms (1.4 pmol) with purified 2B8 monoclonal antibody (stock solution 1 mg/ml), dilution of 2B8 antibody 100,000x - lanes: (1) CA I, (2) CA II, (3) CA VA, (4) CA VB, (5) CA IX, (6) CA XII.
