# Supplementary material for: Inhibitory Effect of Human Anti-CA I Autoantibodies and Development of Monoclonal Antibody mAb 2B8 Targeting Carbonic Anhydrase I
Source: Mediators Inflamm. 2024 Dec 30;2024:9981131. doi: 10.1155/mi/9981131 (PMC11703592; doi:10.1155/mi/9981131)
Supplement: Supporting Information 4 — Figure S3: Dot-blot analysis: immunoreactivity of mAb 2B8 with various forms of CA I: (1) native CA I (5 µg), (2) CA I unfolded by RapiGest and treated with DTT and IAA (5 µg), (3) unfolded CA I digested by TPCK-trypsin (5 µg), (4) BSA (5 µg), and (5) 10 mM phosphate buffer pH 7.0; dilution: 1000x; dilution of secondary HRP-conjugated antibody: 1000x; colorimetric detection: Opti-4CN kit. [file 9981131.f4.pptx]

## Slide 1
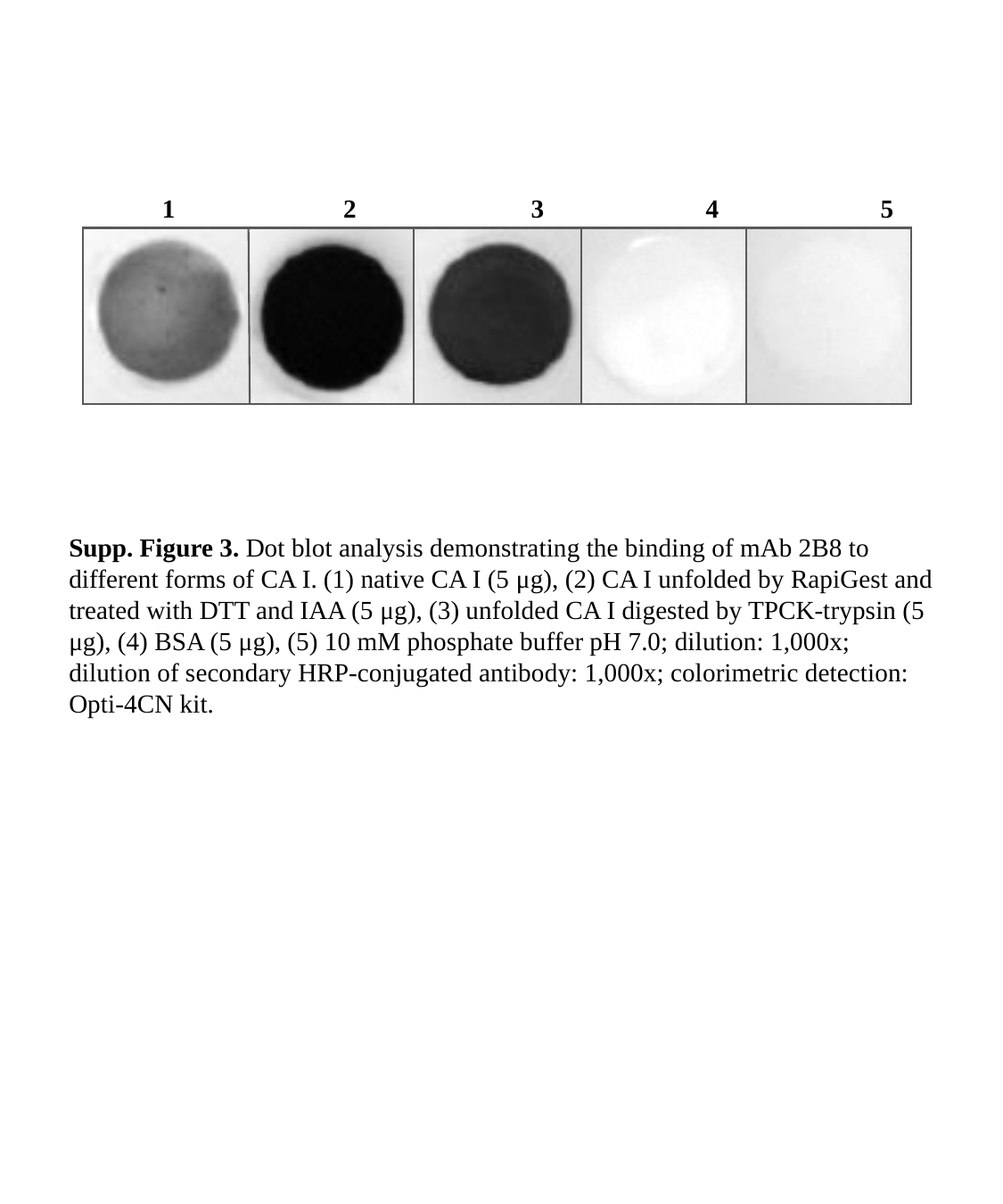

1 2 3 4 5
Supp. Figure 3. Dot blot analysis demonstrating the binding of mAb 2B8 to different forms of CA I. (1) native CA I (5 μg), (2) CA I unfolded by RapiGest and treated with DTT and IAA (5 μg), (3) unfolded CA I digested by TPCK-trypsin (5 μg), (4) BSA (5 μg), (5) 10 mM phosphate buffer pH 7.0; dilution: 1,000x; dilution of secondary HRP-conjugated antibody: 1,000x; colorimetric detection: Opti-4CN kit.
