# Supplementary material for: Inhibitory Effect of Human Anti-CA I Autoantibodies and Development of Monoclonal Antibody mAb 2B8 Targeting Carbonic Anhydrase I
Source: Mediators Inflamm. 2024 Dec 30;2024:9981131. doi: 10.1155/mi/9981131 (PMC11703592; doi:10.1155/mi/9981131)
Supplement: Supporting Information 5 — Figure S4: Coomassie brilliant blue stained SDS–PAGE (10% gel) analysis following immunoprecipitation of (A) native and (B) unfolded CA I (20 µg) using magnetic immunosorbent SeraMag (2 mg) coupled with mAb anti-CA I IgG, clone 2B8 (100 µg)—lanes: (1) molecular markers (Bio-Rad, Hercules, CA, USA), (2) initial fraction of native/unfolded CA I, (3) fraction of CA I after incubation (1.5 h) with immunosorbent, (4) first washing fraction, (5) first elution fraction, (6) second elution fraction, and (7) third elution fraction. [file 9981131.f5.pptx]

## Slide 1
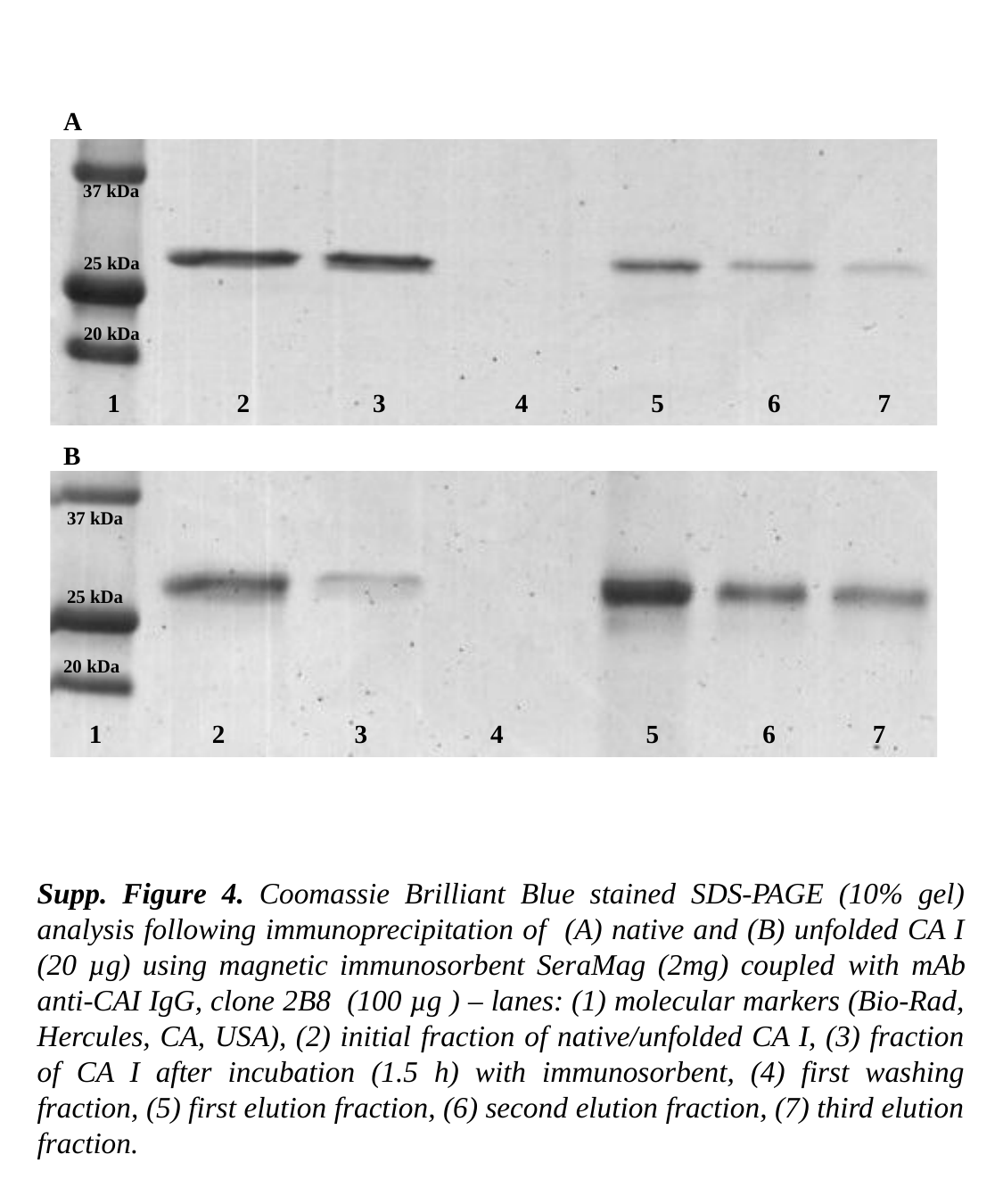

A
37 kDa
25 kDa
20 kDa
 1 2 3 4 5 6 7
B
37 kDa
25 kDa
20 kDa
 1 2 3 4 5 6 7
Supp. Figure 4. Coomassie Brilliant Blue stained SDS-PAGE (10% gel) analysis following immunoprecipitation of (A) native and (B) unfolded CA I (20 µg) using magnetic immunosorbent SeraMag (2mg) coupled with mAb anti-CAI IgG, clone 2B8 (100 µg ) – lanes: (1) molecular markers (Bio-Rad, Hercules, CA, USA), (2) initial fraction of native/unfolded CA I, (3) fraction of CA I after incubation (1.5 h) with immunosorbent, (4) first washing fraction, (5) first elution fraction, (6) second elution fraction, (7) third elution fraction.
