# Supplementary material for: Inhibitory Effect of Human Anti-CA I Autoantibodies and Development of Monoclonal Antibody mAb 2B8 Targeting Carbonic Anhydrase I
Source: Mediators Inflamm. 2024 Dec 30;2024:9981131. doi: 10.1155/mi/9981131 (PMC11703592; doi:10.1155/mi/9981131)
Supplement: Supporting Information 6 — Figure S5: Initial proteomic analysis of PC3 cells (106) incubated for 2 h with 100 µg/ml of mAb 2B8. (A) Principal component analysis (PCA) illustrating the clustering of PC3 cell samples based on proteomic profiles and (B) Venn diagram displaying the number of proteins uniquely identified in PC3 treated cells, the untreated control, and the number of shared proteins. [file 9981131.f6.pptx]

## Slide 1
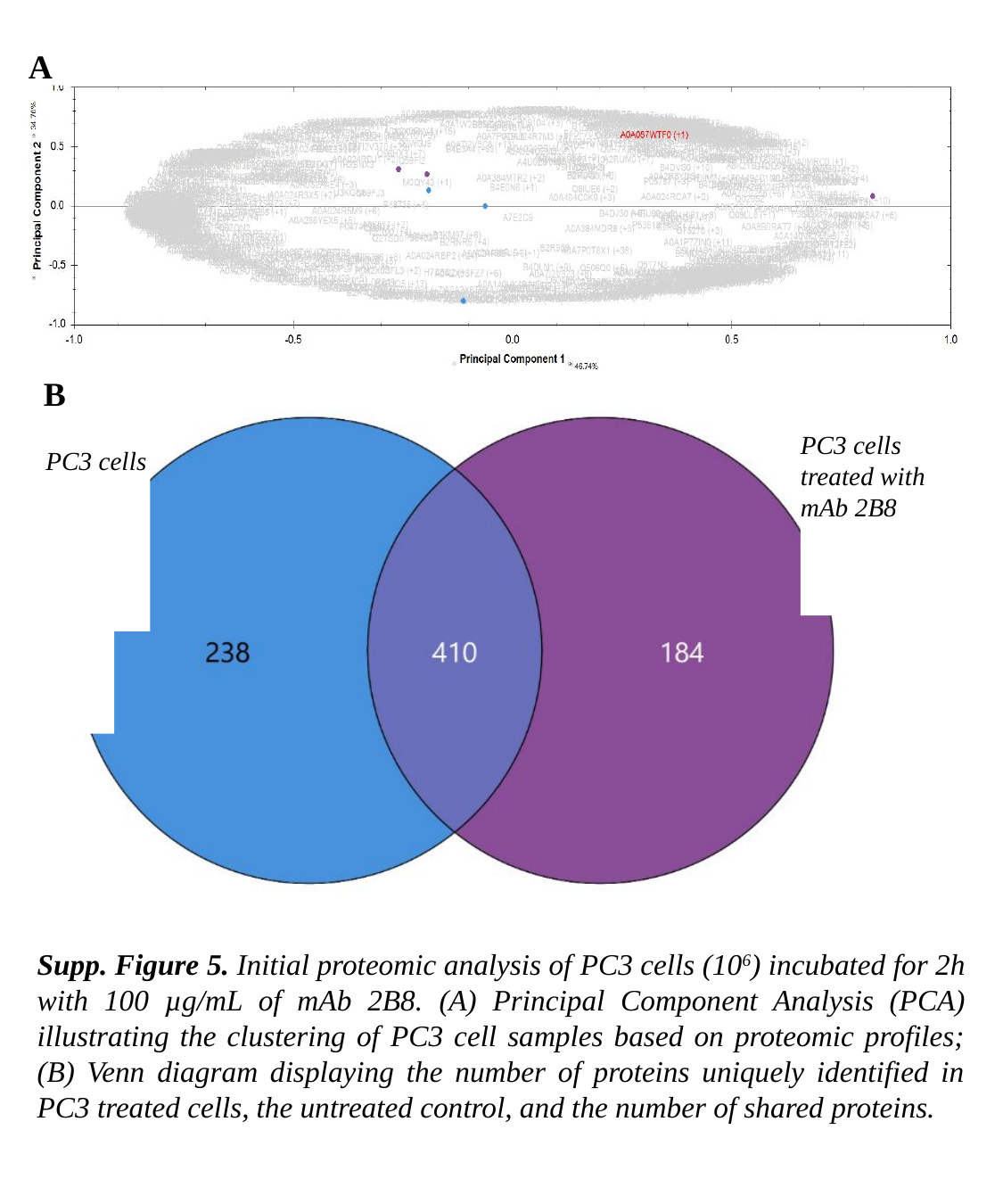

A
B
PC3 cells treated with mAb 2B8
PC3 cells
Supp. Figure 5. Initial proteomic analysis of PC3 cells (106) incubated for 2h with 100 µg/mL of mAb 2B8. (A) Principal Component Analysis (PCA) illustrating the clustering of PC3 cell samples based on proteomic profiles; (B) Venn diagram displaying the number of proteins uniquely identified in PC3 treated cells, the untreated control, and the number of shared proteins.
