# Supplementary material for: Inhibitory Effect of Human Anti-CA I Autoantibodies and Development of Monoclonal Antibody mAb 2B8 Targeting Carbonic Anhydrase I
Source: Mediators Inflamm. 2024 Dec 30;2024:9981131. doi: 10.1155/mi/9981131 (PMC11703592; doi:10.1155/mi/9981131)
Supplement: Supporting Information 7 — Figure S6: The coverage map of CA I showing (A) tryptic peptide fragments detected in the initial sample (73.1% sequence coverage) and (B) tryptic peptide fragments detected in the third elution fraction obtained during the epitope extraction experiment. [file 9981131.f7.pptx]

## Slide 1
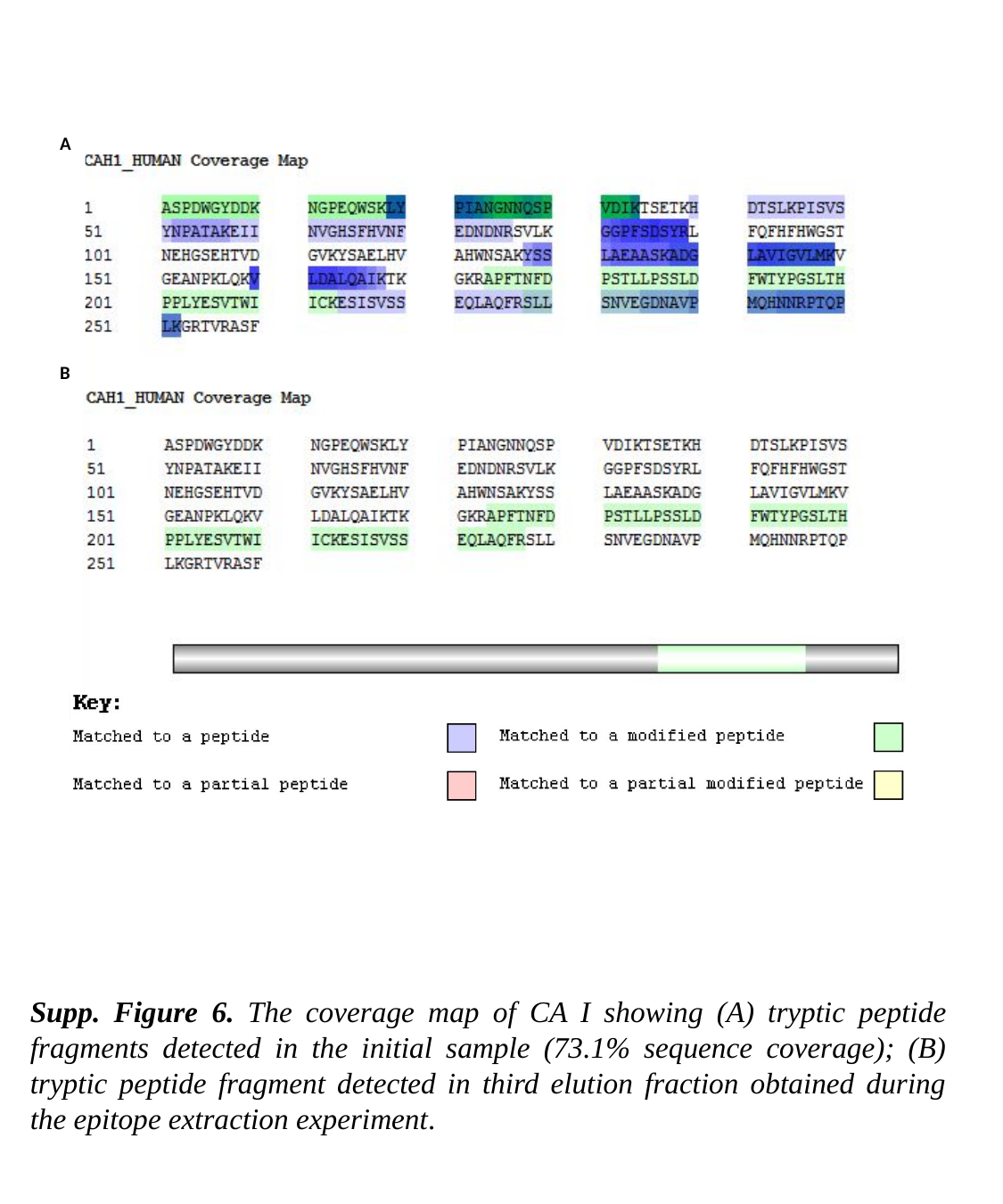

A
B
Supp. Figure 6. The coverage map of CA I showing (A) tryptic peptide fragments detected in the initial sample (73.1% sequence coverage); (B) tryptic peptide fragment detected in third elution fraction obtained during the epitope extraction experiment.
